# Amidofluorene-appended lower rim 1,3-diconjugate of calix[4]arene: synthesis, characterization and highly selective sensor for Cu^2+^

**DOI:** 10.3762/bjoc.12.163

**Published:** 2016-08-04

**Authors:** Rahman Hosseinzadeh, Mohammad Nemati, Reza Zadmard, Maryam Mohadjerani

**Affiliations:** 1 Department of Organic Chemistry, Faculty of Chemistry, University of Mazandaran, Babolsar, Iran; 2 Chemistry and Chemical Engineering Research Center of Iran (CCERCI), Tehran, Iran; 3 Department of Molecular and Cell Biology, Faculty of Basic Science, University of Mazandaran, Babolsar, Iran

**Keywords:** calix[4]arene, chemosensor, copper ions, fluorene, fluorescence

## Abstract

Functionalization of calix[4]arene with amidofluorene moieties at the lower rim led to formation of the 1,3-diconjugate of calix[4]arene **L** as a novel fluorescent chemosensor for Cu^2+^. The receptor molecule **L** exhibited a pronounced selectivity towards Cu^2+^ over other mono and divalent ions. The formation of the complex between **L** and Cu^2+^ was evaluated by absorption, fluorescence and ^1^H NMR spectroscopy. The sensor **L** showed a remarkable color change from colorless to purple and a fluorescence quenching only upon interaction with Cu^2+^. The 1:1 stoichiometry of the obtained complex has been determined by Job’s plot. The association constant determined by fluorescence titration was found to be 1.8 × 10^6^ M^−1^. The sensor showed a linear response toward Cu^2+^ in the concentration range from 1 to 10 µM with a detection limit of 9.6 × 10^−8^ M.

## Introduction

Owing to the substantial role of fluorescent chemosensors in biological, environmental, and chemical processes, their design and synthesis, especially for detection of metal ions has attracted great attention in supramolecular chemistry [1–4]. Copper is one of the crucial biological elements, which presents as catalytic cofactor for a variety of metallo-enzymes such as superoxide dismutase, cytochrome c oxidase, lysyl oxidase and tyrosinase, etc. [5–9]. However, excess amounts of Cu^2+^ are hazardous, due to generate reactive oxygen species (ROS) that disturbs cellular metabolism [10–11]. On the other hand, its deficiency may cause haematological and neurological diseases [12]. Therefore, the selective and sensitive measurement of Cu^2+^ ions – either as significant environmental pollutant or an essential trace element in human body – is a great challenge and the development of synthetic receptors based on organic ligands for the detection of Cu^2+^ seems to be necessary [13]. Accordingly, during the last decades, synthesis and modification of chemosensors based on supramolecular structures, especially calixarene derivatives, has been an appealing field for research [14]. Calixarene has been considered as an effective molecular scaffold in the improvement of fluorescent and chromogenic sensors, especially for metal-ion recognition [14–15]. Calix[4]arene derivatives having different binding centers such as nitrogen, oxygen and sulfur sites for recognition of metal ions can be readily synthesized [14,16–17]. A number of calix[4]arene-based fluorescence sensors for copper ions have been reported in the literature [18–27], including calix[4]arene bearing anthraceneisoxazolymethyl [3], quinolone [28], 5-nitrosalicylaldehyde [29], 3-alkoxy-2-naphthoic acid [30], coumarine [31] and benzothiazole [10,15] groups.

**Scheme 1:**
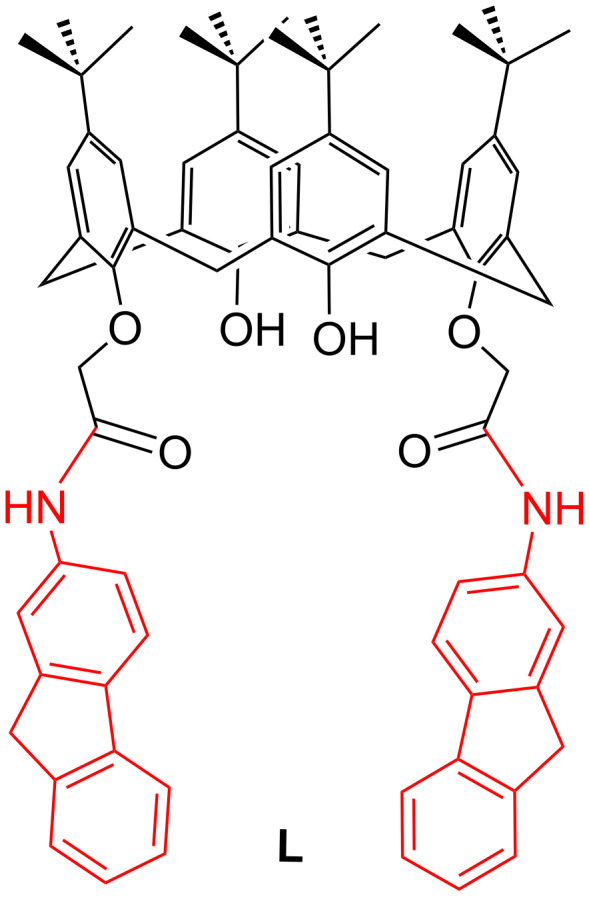
Fluorene appended 1,3-diconjugate of calix[4]arene.

In continuation of our studies on developing novel chemosensors containing fluorenyl moieties as fluorogenic group [32–34], we report here the design and synthesis of a new fluorene-appended 1,3-diconjugate of calix[4]arene (**L**), which possess distal amide groups on the lower rim of the conical framework to recognize Cu^2+^ with high sensitivity and specificity (Scheme 1).

## Results and Discussion

### Synthesis

**Scheme 2:**
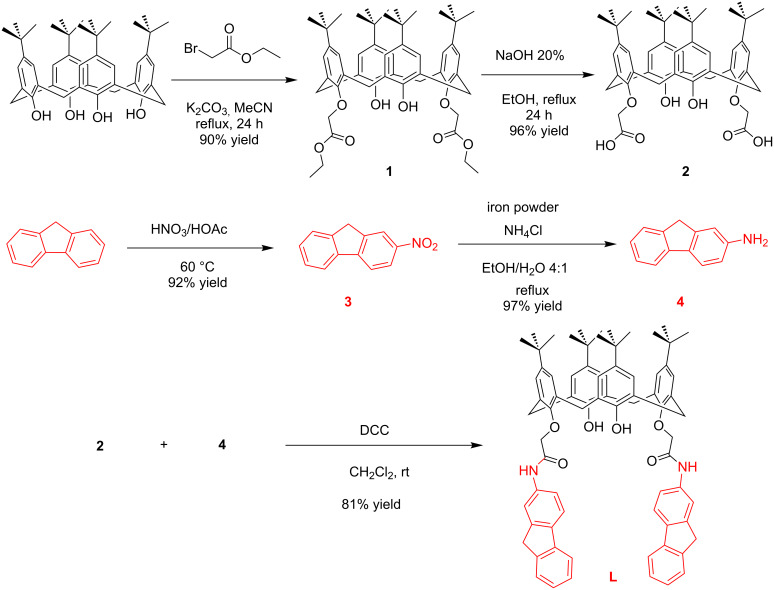
Synthesis route of fluorene-appended amido-linked 1,3-diconjugate of calix[4]arene **L**.

The synthesis of 5,11,17,23-tetra-*tert*-butyl-25,27-di[(9*H*-fluoren-2-yl)aminocarbonylmethoxy]-26,28-dihydroxycalix[4]arene (**L**) is depicted in Scheme 1. The synthesis began with the reaction of *p*-*tert*-butylcalix[4]arene and ethyl bromoacetate in the presence of potassium carbonate in acetonitrile. Ester hydrolysis of compound **1** afforded the 5,11,17,23-tetra-*tert*-butyl-25,27-di(hydroxycarbonylmethoxy)-26-28-dihydroxy calix[4]arene (**2**) in high yield. Coupling of this diacid **2** with 9*H*-fluoren-2-amine (**4**, obtained from nitration of fluorene and reduction of the resulting 2-nitrofluorene, (**3**)) in the presence of *N*,*N*-dicyclohexylcarbodiimide (DCC) in dichloromethane at room temperature gave the desired receptor **L** in 81% yield (Scheme 2). All the compounds including receptor **L** has been characterized by various spectral techniques such as ^1^H, ^13^C NMR and high resolution mass spectrometry (HRMS) (see Supporting Information File 1, Figures S1–S11).

### General procedure for UV–vis experiments

The evaluation of cation-ligand interaction was performed with UV–vis spectroscopy. The UV–vis measurement of receptor **L** in CH_3_CN exhibited three absorption peaks at 282, 290, and 314 nm. Furthermore, the variation of UV–vis spectra was monitored by adding 10 equivalents of different metal ions as perchlorate salts (Cu^2+^, Hg^2+^, Pb^2+^, Zn^2+^,Co^2+^, Ni^2+^, Cd^2+^, Ag^+^, Ba^2+^, K^+^, Na^+^ and Li^+^).

**Figure 1:**
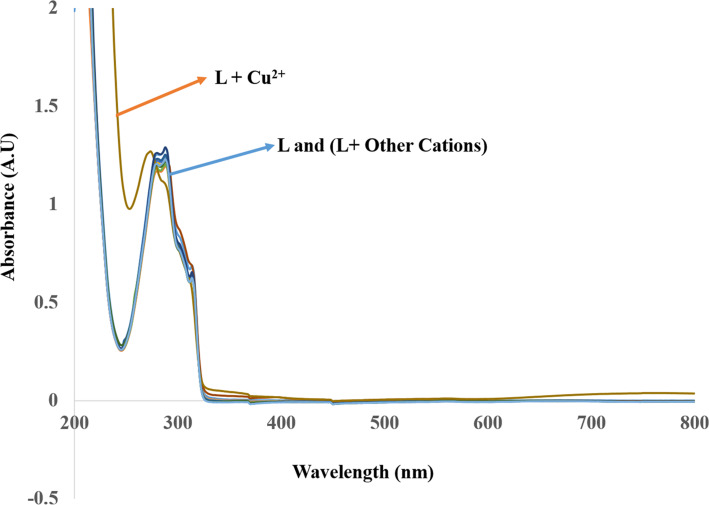
Absorption spectra of **L** (1.0 × 10^−5^ M) and its complexes with different metals (10 equiv) in MeCN.

**Figure 2:**
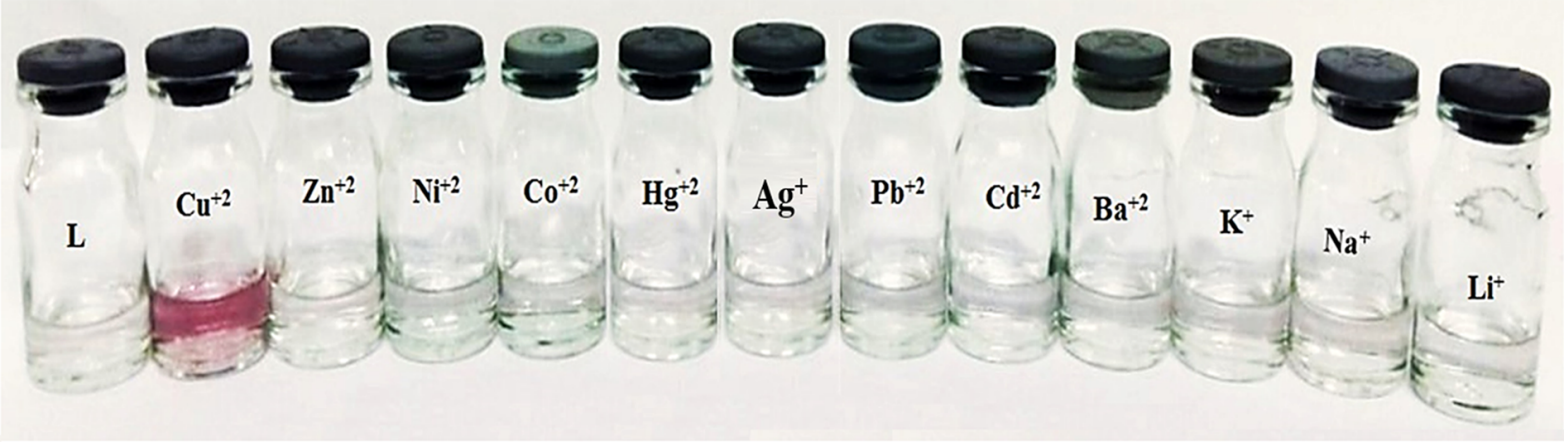
Color changes of receptor **L** upon addition of 10 equiv of various metal ions as their perchlorate salts.

Unlike Cu^2+^ ions, the addition of 10 equiv of other metal ions to the 1.0 × 10^−5^ M solutions of **L** resulted in no significant changes in the absorption spectra of ligand **L**. However, addition of Cu^2+^ to **L** resulted in a blue shift of the absorption band in the area of 280–290 nm (see Supporting Information File 1, Figure S12). Furthermore, a weak and broad absorption band from 600–800 nm was also observed (Figure 1). As shown in Figure 2, there was an evident color change from colorless to purple, which could be observed by the naked eye.

**Figure 3:**
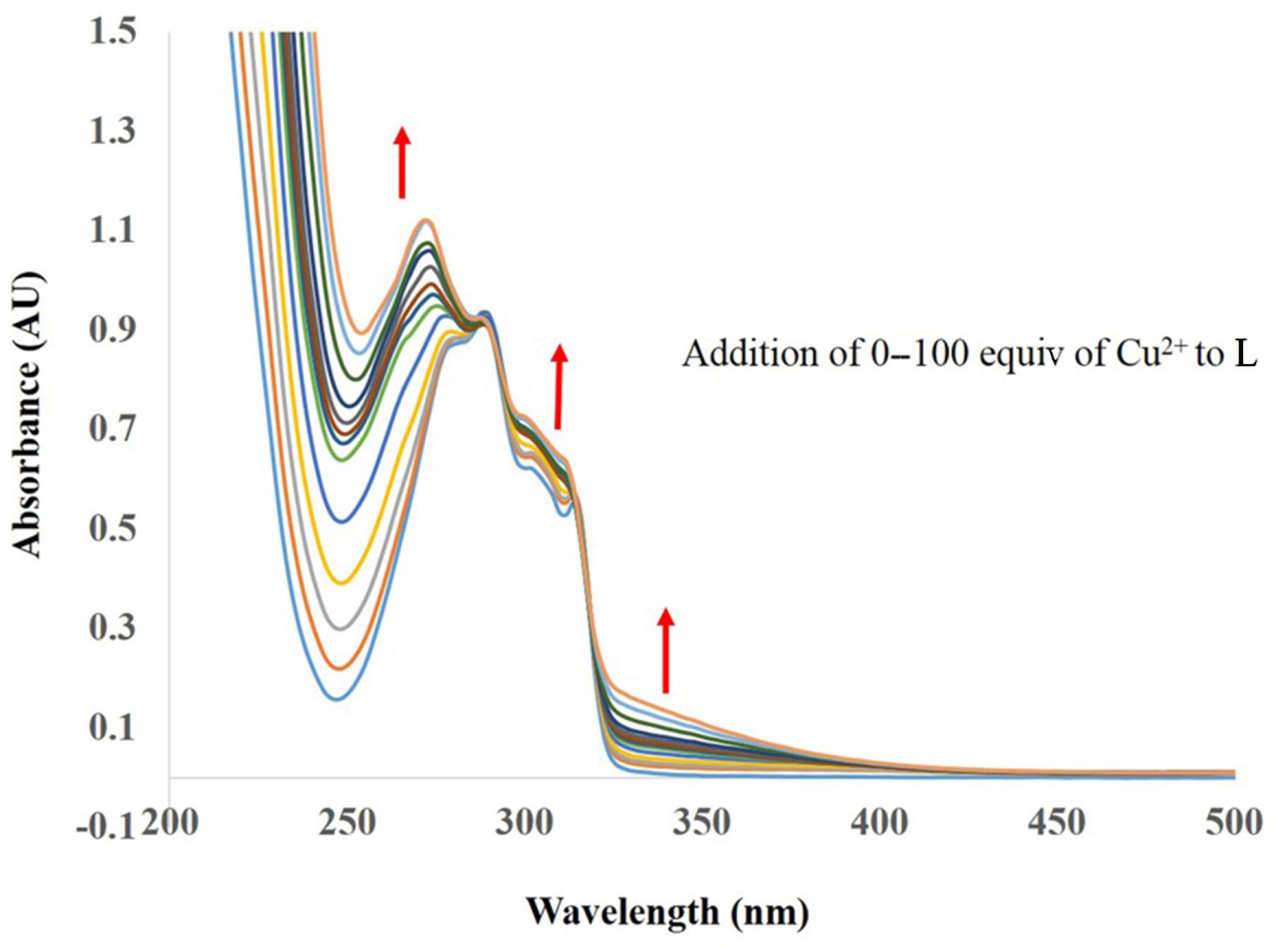
Influence of the addition of increasing amounts (0–100 equiv) of Cu^2+^ on the absorption spectra of **L** (1.0 × 10^−5^ M) in MeCN.

The selectivity of receptor **L** towards Cu^2+^ was precisely assessed by UV–vis spectroscopy during titration with different concentrations of Cu^2+^ from 0 to 100 equivalents in CH_3_CN. In the absence of Cu^2+^ ions, the ligand **L** exhibited three absorption bands at ≈282, ≈290 and ≈314 nm (Figure 3). Upon addition of Cu^2+^, the absorption band at 282 nm showed a blue shift along with an increase in peak intensity. The two observed isosbestic points at 295 and 318 nm upon addition of Cu^2+^ revealed formation of a stable complex between **L** and the copper ion. Moreover, upon addition of Cu^2+^, at higher concentrations of **L** a broad signal appeared at the area of 600–800 nm which can be related to a d→d transition (see Supporting Information File 1, Figure S13). This result is in accordance with those reported in literature [10] and indicates that the **L** binds copper as Cu^2+^.

### Fluorescence titrations of **L** with metal ions

**Figure 4:**
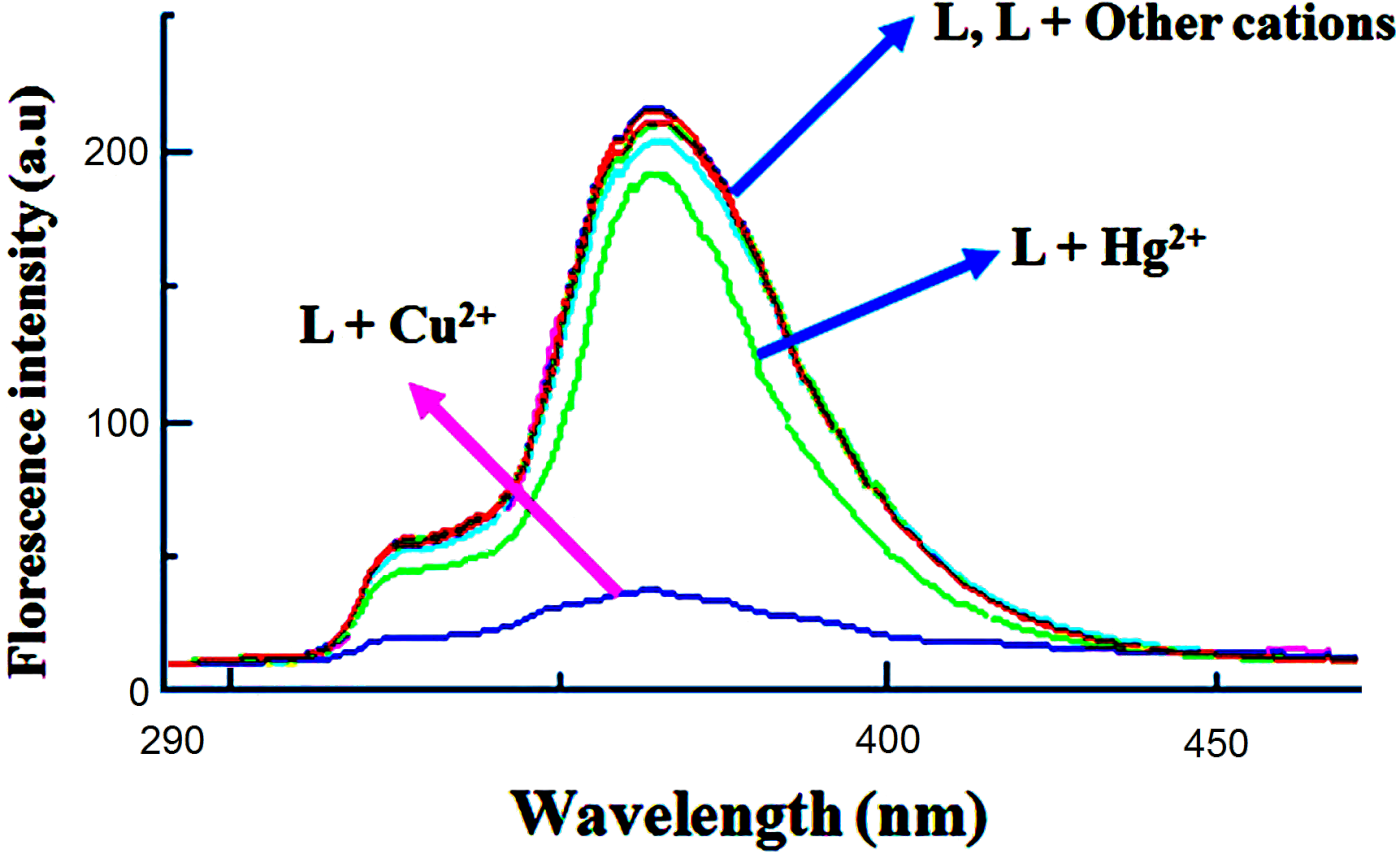
Fluorescence spectra of **L** (1.0 × 10^−5^ M) in MeCN upon addition of different metal ions (10 equiv) with an excitation at 280 nm.

Fluorescence spectroscopy was applied to investigate the recognition properties of chemosensor **L** toward various metal ions in MeCN. In the absence of metal ions, the receptor **L** represents fluorescence emission at ≈365 nm when excited at 280 nm. Upon addition 10 equiv of Cu^2+^ to the MeCN solution of sensor **L**, a remarkable fluorescence quenching was observed (Figure 4). In order to explore the selectivity of sensor **L**, similar experiments were carried out in the presence of other perchlorate salts of Cu^2+^, Hg^2+^, Pb^2+^, Zn^2+^, Co^2+^, Ni^2+^, Cd^2+^, Ag^+^, Ba^2+^, K^+^, Na^+^ and Li^+^. The fluorescence spectra showed almost no obvious change relative to the free ligand **L** (Figure 4).

**Figure 5:**
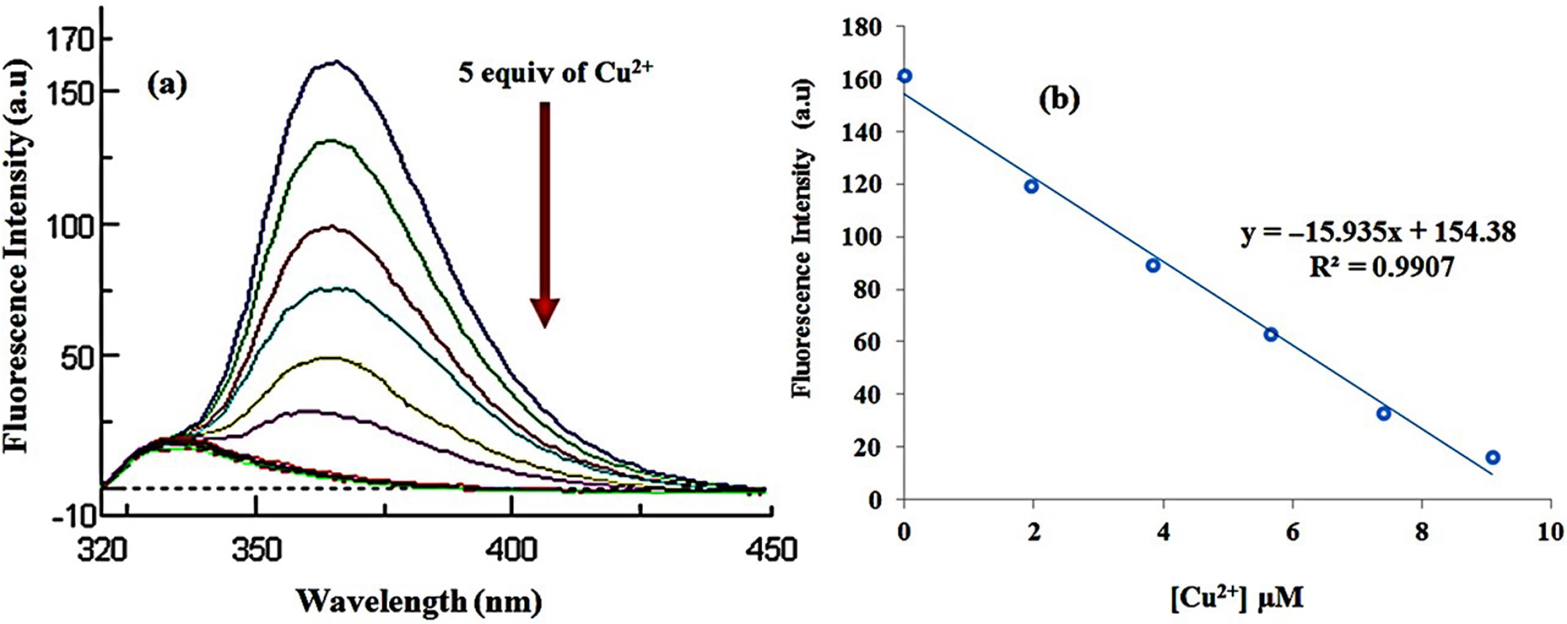
(a) Fluorescence spectra (1.0 × 10^−5^ M) of **L** recorded upon the addition of copper ion (0–5 equiv) in MeCN; excitation at 280 nm and emission at 365 nm. (b) Calibration curve of fluorescence intensity of **L** with Cu^2+^ ion concentrations.

Titration of **L** with Cu^2+^ resulted in a gradual quenching of the fluorescence emission at the 365 nm band as a function of increasing Cu^2+^ concentration. The fluorescence intensity spectrum showed a gradual decrease up to 5 equiv of Cu^2+^. With higher Cu^2+^ concentrations the fluorescence quenched completely (Figure 5a). The stoichiometry of the complex was found to be 1:1 between **L** and Cu^2+^ based on the Job’s plot and its association constant was calculated using the Benesi–Hildebrand equation [35] to be 1.8 × 10^6^ M^−1^ (see Supporting Information File 1, Figures S14 and S15). From these results it can be concluded that the Cu^2+^ ion has a preferential interaction with receptor **L**. It can be concluded from the results displayed in Figure 5b that the fluorescence quenching intensity at 365 nm has a linear relationship with Cu^2+^ concentration. The fluorescent response of **L** toward Cu^2+^ has a linear dynamic range from 1 to 10 µM with a detection limit of 9.6 × 10^−8^ M (see Supporting Information File 1, Figure S16).

### Competitive metal ion titrations

**Figure 6:**
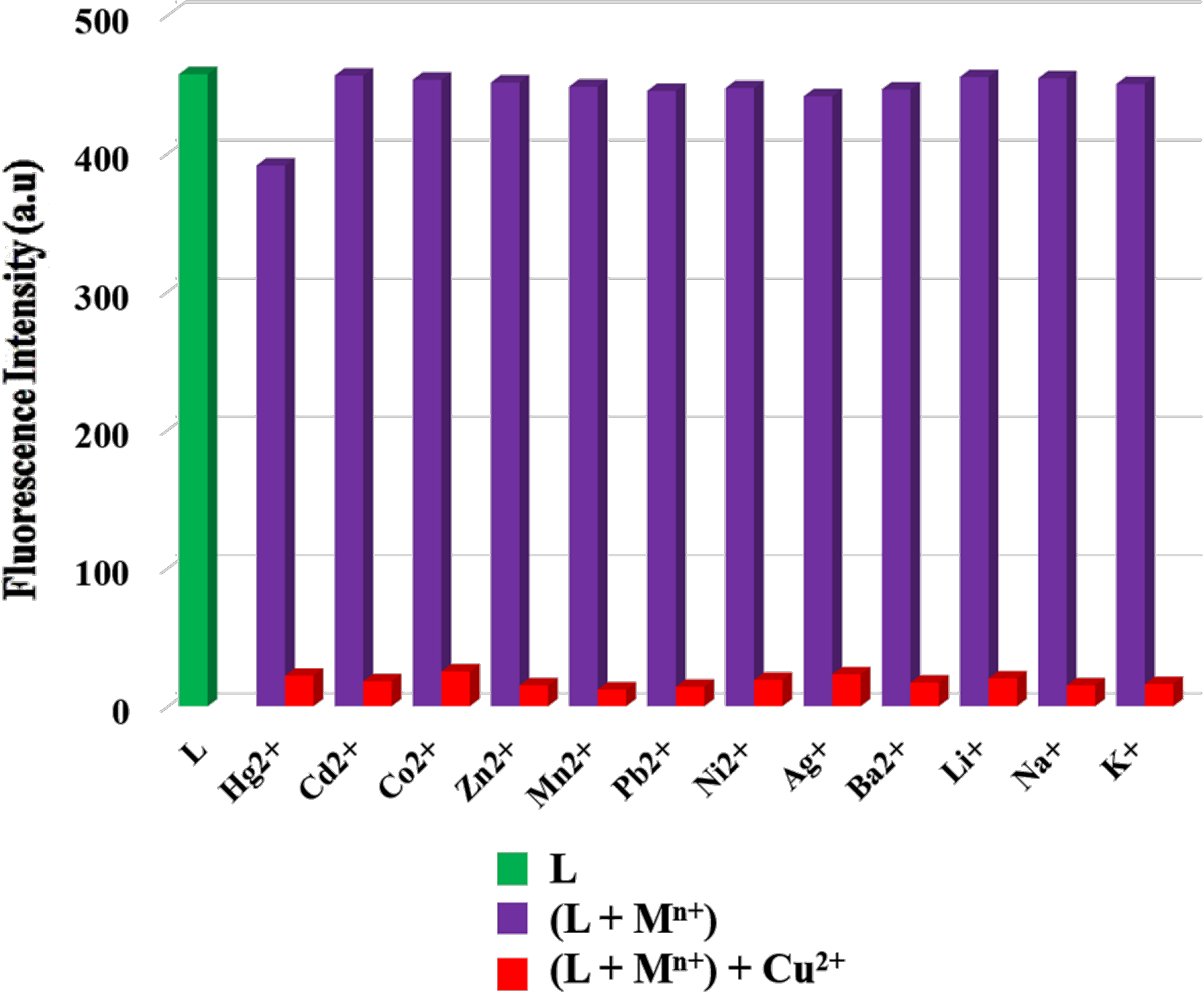
Fluorescence intensity of **L** (1.0 × 10^−5^ M) upon the addition of 10 equiv Cu^2+^ in the presence of 10 equiv of other metal ions (as perchlorate salts) in MeCN.

In order to examine the competitive recognition of Cu^2+^ by sensor **L**, the effect of Cu^2+^ was studied in the presence of other metal ions. The results showed that the fluorescence emission intensity of [**L** + M*^n^*^+^] is altered in the presence of Cu^2+^ ions (Figure 6). It is noteworthy that complex [**L** + M*^n^*^+^] exhibited no considerable fluorescence variations in comparison with **L**, and addition of Cu^2+^ ions to the corresponding solutions resulted in fluorescence quenching. Therefore, **L** can recognize Cu^2+^ even in the presence of other metal ions in acetonitrile solution.

### ^1^H NMR titration of **L** with Cu^2+^

**Figure 7:**
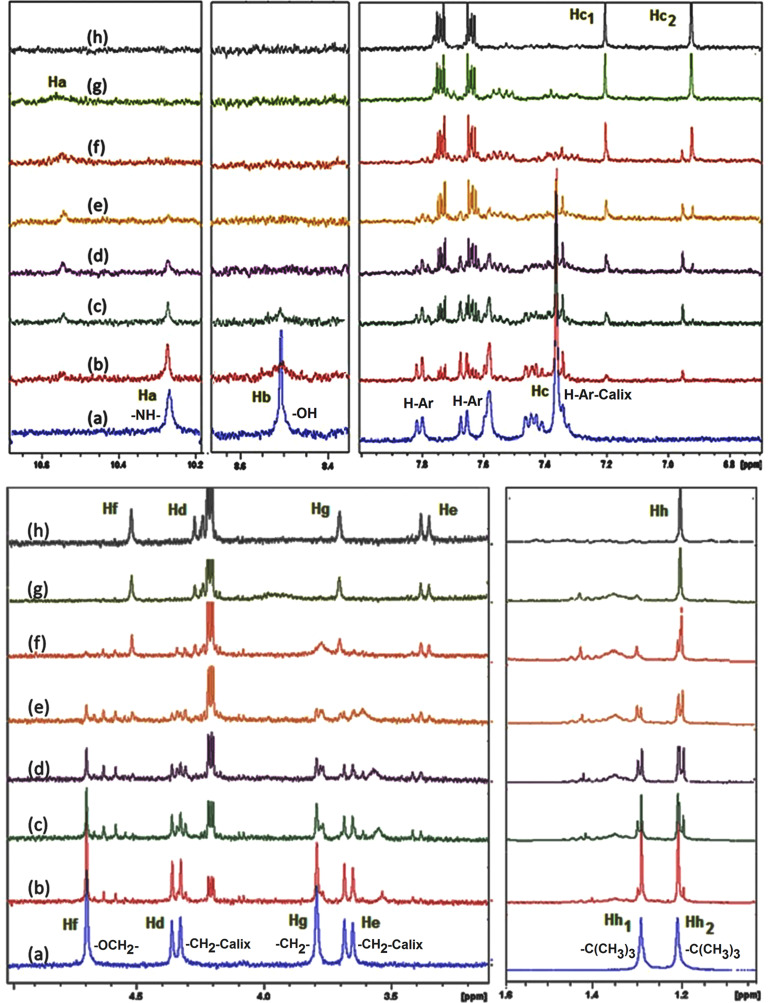
^1^ H NMR (400 MHz, CD_3_CN) spectra of **L** upon addition of (a) 0.0 equiv, (b) 0.2 equiv, (c) 0.4 equiv, (d) 0.6 equiv, (e) 0.8 equiv, (f) 1.0 equiv, (g) 1.2 equiv and (h) 1.5 equiv of Cu^2+^.

In order to understand the mode of complexation of **L** with Cu^2+^, ^1^H NMR titrations were carried out (Figure 7). Upon the initial addition of Cu^2+^ into the CD_3_CN solution of **L**, signals related to OH phenolic groups (Hb) and amidic protons (Ha) were first broadened and then finally disappeared. Furthermore, the signals of Ar–CH_2_ (Hd, He) and –O–CH_2_ (Hf) porotons, which are close to the binding site of receptor **L** were affected and downfield shifted by the complexation of **L** with Cu^2+^. A detailed analysis of the ^1^H NMR spectra reveals the significant changes of almost all the other proton signals in the ligand. For instance, the peak of aromatic ring hydrogen atoms (Hc, resonated at 7.35 ppm) was converted to two distinct peaks at 7.20 and 6.95 ppm (Hc_1_, Hc_2_). In addition, signals belonging to the fluorene moieties and *tert*-butyl groups, which are quite far away from binding site, were also affected by complex formation. For example, the peaks of the *tert*-butyl groups of receptor **L** resonated at 1.29 (Hh_1_) and 1.22 ppm (Hh_2_) turned to one signal at 1.20 ppm during the titration.

**Scheme 3:**
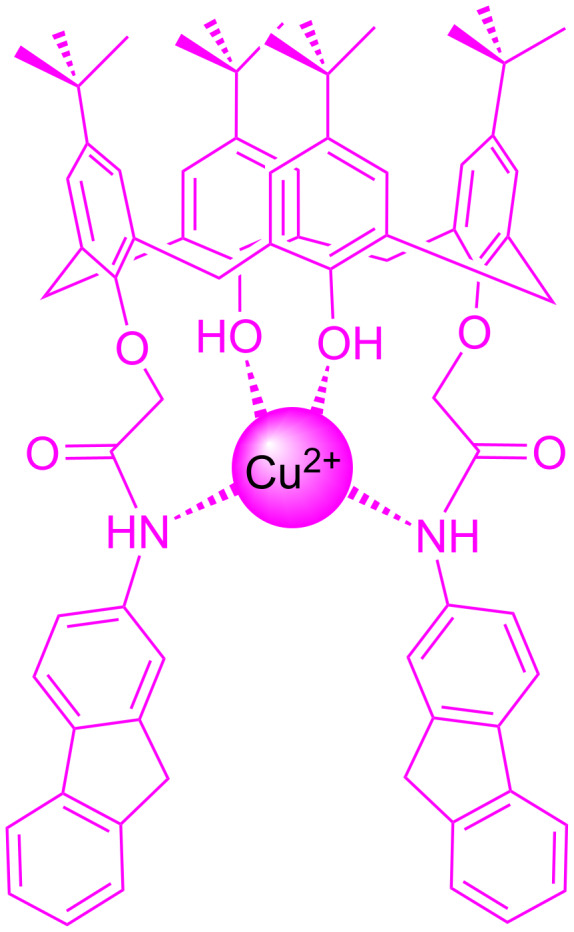
A proposed binding mode between **L** and Cu^2+^.

All these observations might be attributed to the changes in electron density, the anisotropy effect and the changes in the conformation of receptor **L** during the complex formation. The fact that receptor **L** shows a sensitive recognition affinity to copper ions and a much more obvious shift of ^1^H NMR peaks in the presence of Cu^2+^ is actually not surprising because of a relatively rigid binding pocket of receptor **L** providing four sites of NH and OH which allows a suitable complex formation with a copper ion (Scheme 3).

To show the high selectivity of chemosensor **L** for copper ions, the association constant and limit of detection (LOD) were compared with those reported for other related chemosensors (Table 1). It should be emphasized that due to different conditions applied to measure these values, we could not compare properly the performance of chemosensor **L** with those reported in literature. However, the associating constants of ligands having two moieties at the lower rim of a calix[4]arene scaffold were compared with the present chemosensor. The results indicated that the synthesized chemosensor **L** has a lower LOD and a higher *K*_a_ in comparison with other chemosensors, which shows a stable and selective complexation with Cu^2+^.

**Table 1:** Comparison of lower rim 1,3-diconjugate derivatives of calix[4]arene with chemosensor **L**.

Ligand	*K*_a_ (M)	LOD (M^−1^)

benzothiazole [15]	18893 ± 1200	403 (ppb)
2-picolyl [36]	30221 ± 1600	196 ppb
arylisoxazole [3]	1.58 × 10^4^	–
benzimidazole [10]	7.24 × 10^9^	96 ppb
fluorene (this work)	1.8 × 10^6^	96 ppb

## Conclusion

In this study, the amidoflourene-appended calix[4]arene 1,3-diconjugated **L** was synthesized as an efficient colorimetric and fluorescent probe for Cu^2+^ detection. The sensitivity and selectivity of chemosensor **L** toward Cu^2+^ has been investigated by spectroscopic methods. This sensor has a high affinity (*K*_a_ = 1.8 × 10^6^ M^−1^) for copper ions. The competitive metal-ion titrations by fluorescence spectroscopy showed that Cu^2+^ can be selectively detected in the presence of other common metal ions. It is noteworthy to mention that the chemosensor **L** can be used as a “naked eye” indicator for Cu^2+^. Furthermore, fluorescence quenching of **L** upon addition of Cu^2+^ showed a detection limit of about 9.6 × 10^−8^ mol/L in the concentration range from 1 to 10 µM. This result demonstrated the possibility of quantitative detection of low levels of Cu^2+^ in MeCN by using this chemosensor.

## Experimental

### Instruments and reagents

^1^H and ^13^C NMR spectra were recorded at 400 and 100 MHz, respectively, on a Bruker Avance III 400 MHz spectrometer. CDCl_3_ and CD_3_CN were used as NMR solvents and TMS as internal standard. High-resolution mass spectra (HRMS) were recorded on a Qstar ESI-q-TOF mass spectrometer (Applied Biosystems, Darmstadt, Germany). SPEKOL 2000 Analytik Jena spectrometer were used for recording of UV–vis spectra in acetonitrile as solvent. Fluorescence spectra were recorded on a JASCO FP-6500 spectrophotometer. All chemicals used in this paper including metal salts were purchased from Merck Company and were used without further purifications. Column chromatography was performed on silica gel (200–400 mesh). As starting material, *p*-*tert*-butylcalix[4]arene was synthesized according to the reported literature procedure [37].

**5,11,17,23-Tetra-*tert*-butyl-25,27-di(ethoxycarbonylmethoxy)-26,28-dihydroxycalix[4]arene (1)** [38]: A mixture of *p*-*tert*-butylcalix[4]arene (2.0 g, 3.08 mmol) and potassium carbonate (1.0 g, 7.3 mmol) in dry acetonitrile (50 mL) was stirred. Ethyl bromoacetate (1 mL, 9 mmol) was added. The mixture was heated under reflux for 24 h. It was then filtered and the residue was washed with dichloromethane (2 × 50 mL). The solvents were removed from the filtrate under vacuum. Recrystallization of the resulting solid from dichloromethane/methanol gave 5,11,17,23-tetra-*tert*-butyl-25,27-diethoxycarbonylmethoxy-26,28-dihydroxycalix[4]arene (2.3 g, 90%). ^1^H NMR (CDCl_3_, 400 MHz) δ 7.10 (s, 2H, ArOH), 7.05 (s, 4H, ArH), 6.84 (s, 4H, ArH), 4.75 (s, 4H, O-CH_2_), 4.48 (d, *J* = 13.2 Hz, 4H, Ar-CH_2_-Ar), 4.32 (q, *J* = 7.2 Hz, 4H, -CH_2_CH_3_), 3.35 (d, *J* = 13.2 Hz, 4H, Ar-CH_2_-Ar), 1.35 (t, *J* = 7.2 Hz, 6H, CH_2_CH_3_), 1.29 (s, 18H, -C(CH_3_)_3_), 1.01 (s, 18H, C(CH_3_)_3_) ppm; ^13^C NMR (CDCl_3_, 100 MHz) δ 169.28, 150.73, 150.29, 147.07, 141.47, 132.52, 127.97, 125.76, 125.08, 72.41, 61.27, 33.94, 33.82, 31.88, 31.66, 31.05, 14.17 ppm.

**5,11,17,23-Tetra-*tert*-butyl-25,27-di(hydroxycarbonylmethoxy)-26,28-dihydroxycalix[4]arene (2)** [38]: A mixture of 5,11,17,23-tetra-*tert*-butyl-25,27-di(ethoxycarbonylmethoxy)-26,28-dihydroxycalix[4]arene (**1**, 1.0 g, 1.2 mmol) and 20% aqueous sodium hydroxide (2 mL, 1.2 mmol) in ethanol (50 mL) was refluxed for 24 h. The residue was diluted with water (50 mL). HCl (3 mol·L^−1^) was added to the resulting suspension until pH 1 was reached. This suspension was washed with chloroform (100 mL) and brine (30 mL). The organic layer was dried over MgSO_4_ and the solvents were removed under vacuum to give 5,11,17,23-tetra-*tert*-butyl-25,27-di(hydroxycarbonylmethoxy)-26,28-dihydroxycalix[4]arene (**2**, 0.9 g, 96%). ^1^H NMR (CDCl_3_, 400 MHz) δ 7.09 (s, 4H, ArH) , 6.98 (s, 4H, ArH), 6.9–5.4 (bs, 4H, -OH, -COOH), 4.71 (s, 4H, O-CH_2_), 4.17 (d, *J* = 13.6 Hz, 4H, Ar-CH_2_-Ar), 3.46 (d, *J* = 13.6 Hz, 4H, Ar-CH_2_-Ar), 1.28 (s, 18H, -C(CH_3_)_3_), 1.09 (s, 18H, C(CH_3_)_3_) ppm; ^13^C NMR (CDCl_3_, 100 MHz) δ 170.32, 149.07, 149.01, 148.46, 143.14, 132.51, 127.25, 126.31, 125.68, 72.42, 34.18, 33.91, 32.32, 31.58, 31.03 ppm.

**2-Nitro-9*H*-fluorene (3)** [39]: 9*H*-Fluorene (6.0 g, 36.1 mmol) was dissolved in 100 mL of glacial acetic acid at 60 °C. 15 mL of nitric acid (65%) were added dropwise (~10 min) at 60 °C upon vigorous stirring. After the addition was completed, the resulting mixture was further stirred at 60 °C. The reaction was monitored by TLC (solvent EtOAc–*n*-heptane 1:9). After appearance of the spot of the dinitro product (≈100 min, *R*_f_ ≈25%), the mixture was poured into 600 mL of water. The resulting crude product was filtered off, washed with water and recrystallized from 200 mL acetonitrile to give compound **3** (7.1 g, 92%) as slightly-yellow needles. ^1^H NMR (CDCl_3_, 400 MHz) δ 8.42 (s, 1H), 8.31 (d, *J* = 8.4 Hz, 1H), 7.89–7.87 (m, 2H), 7.64 (d, *J* = 6.4 Hz, 1H), 7.49–7.43 (m, 2H), 4.02 (s, 2H) ppm; ^13^C NMR (CDCl_3_, 100 MHz) δ 148.09, 146.79, 144.81, 143.92, 139.47, 128.87, 127.43, 125.43, 123.14, 121.34, 120.49, 119.88, 36.96 ppm.

**9*H*-Fluoren-2-amine (4)** [40]: A mixture of 2-nitro-9*H*-fluorene (2.0 g, 9.5 mmol), iron powder (1.0 g, 18.7 mmol), and NH_4_Cl (0.75 g, 12.46 mmol) was refluxed in aqueous ethanol (75 mL of alcohol and 25 mL of water) at 85 °C for 4 h under argon atmosphere. The reaction was monitored by TLC (solvent EtOAc–hexane 2:3). After completion of the reaction, the resulting mixture was treated with 50 mL of saturated aqueous sodium bicarbonate solution and filtered off. The transparent filtrate was concentrated in vacuum in order to remove the organic solvent. The residue was filtered off to yield compound **4** (1.67g, 97%) as transparent plates. The crude product was used directly in the next step. ^1^H NMR (CDCl_3_, 400 MHz) δ 7.66 (d, *J* = 7.6 Hz, 1H), 7.60 (d, *J* = 8.0 Hz, 1H), 7.50 (d, *J =* 7.2 Hz, 1H), 7.35 (td, *J* = 7.6 and *J* = 0.8 Hz, 1H), 7.22 (td, *J* = 7.6 and *J* = 1.2, 1H), 6.9 (br s, 1H), 6.73 (dd, *J* = 8.0 and *J* = 2.4 Hz, 1H), 3.84 (s, 2H), 3.70 (br s, 2H, NH_2_) ppm; ^13^C NMR (CDCl_3_, 100 MHz) δ 145.75, 145.16, 142.27, 142.15, 133.01, 126.64, 125.09, 124.76, 120.67, 118.6, 113.98, 111.82, 36.83 ppm.

**5,11,17,23-Tetra-*tert*-butyl-25,27-di[(9H-fluoren-2-yl)aminocarbonylmethoxy]-26,28-dihydroxycalix[4]arene (L):** A mixture of 5,11,17,23-tetra-*tert*-butyl-25,27-di(hydroxycarbonylmethoxy)-26,28-dihydroxycalix[4]arene (**2**, 0.50 g, 0.65 mmol) and *N,N’*-dicyclohexylcarbodiimide (2.6 mmol) was dissolved in 5 mL dichloromethane and stirred for 10 min at room temperature. 9*H*-Fluoren-2-amine (**4**, 0.20 g, 1.1 mmol) was added and the mixture was stirred for 24 h at room temperature. Then, the solvent was removed and the residue was dissolved in chloroform (50 mL) and washed with water (30 mL). The crude product was purified by silica gel column chromatography using petroleum ether–ethylacetate 3:1 as eluent to give **L** (0.58 g, 81%) as white powder. Mp: 241–243 °C; ^1^H NMR (CDCl_3_, 400 MHz) δ 10.12 (s, 2H, NH), 7.98 (s, 2H, ArOH), 7.73 (d, *J* = 7.6 Hz, 2H, ArH), 7.63 (d, *J* = 0.8 Hz, 2H, ArH), 7.51–7.48 (m, 4H, ArH), 7.41–7.37 (m, 2H, ArH), 7.34–7.3 (m, 4H, ArH), 7.19 (s, 4H, ArH), 7.01 (s, 4H, ArH), 4.7 (s, 4H, O-CH_2_), 4.3 (d, *J* = 13.6 Hz, 4H, Ar-CH_2_-Ar), 3.57 (d, *J* = 13.6 Hz, 4H, Ar-CH_2_-Ar), 3.56 (s, 4H, Ph-CH_2_-Ph), 1.33 (s, 18H, C(CH_3_)_3_), 1.08 (s, 18H, C(CH_3_)_3_) ppm; ^13^C NMR (CDCl_3_, 100 MHz) δ 165.19, 149.53, 148.85, 148.26, 143.92, 143.60, 143.34, 141.52, 137.80, 136.16, 132.17, 127.36, 126.65, 126.40, 126.08, 125.76, 124.87, 119.62, 119.57, 117.43, 115.89, 74.84, 36.85, 34.22, 34.01, 32.14, 31.62, 30.98 ppm. HRMS *m*/*z*: [M]^+^ calcd for C_74_H_78_N_2_O_6_, 1090.5860; found, 1090.4344.

## Supporting Information

File 1 ^1^H NMR and ^13^C NMR spectra of compounds **1**, **2**, **3, 4** and **L**, HRMS of **L**, UV–vis and fluorescene titration spectra of **L** with Cu^2+^ ion solutions.
