# Supplementary material for: Amidofluorene-appended lower rim 1,3-diconjugate of calix[4]arene: synthesis, characterization and highly selective sensor for Cu2+
Source: Beilstein J Org Chem. 2016 Aug 4;12:1749–57. doi: 10.3762/bjoc.12.163 (PMC4979684; doi:10.3762/bjoc.12.163)
Supplement: File 1 — 1H NMR and 13C NMR spectra of compounds 1, 2, 3, 4 and L, HRMS of L, UV–vis and fluorescene titration spectra of L with Cu2+ ion solutions. [file Beilstein_J_Org_Chem-12-1749-s001.pdf]

Supporting Information

for

Amidofluorene-appended lower rim 1,3-diconjugate of calix[4]arene: synthesis, characterization and highly selective sensor for Cu^{2+}

Rahman Hosseinzadeh*[1], Mohammad Nemati[1], Reza Zadmard[2], Maryam Mohadjerani[3]

Address: [1]Department of Organic Chemistry, Faculty of Chemistry, University of Mazandaran, Babolsar, Iran, [2]Chemistry and Chemical Engineering Research Center of Iran (CCERCI), Tehran, Iran and [3]Department of Molecular and Cell Biology, Faculty of Basic Science, University of Mazandaran, Babolsar, Iran

Email: Rahman Hosseinzadeh - r.hosseinzadeh@umz.ac.ir

*Corresponding author

^1H NMR and ^{13}C NMR spectra of compounds 1, 2, 3, 4 and L, HRMS of L, UV–vis and fluorescene titration spectra of L with Cu^{2+} ion solutions

Spectroscopic and analytical data

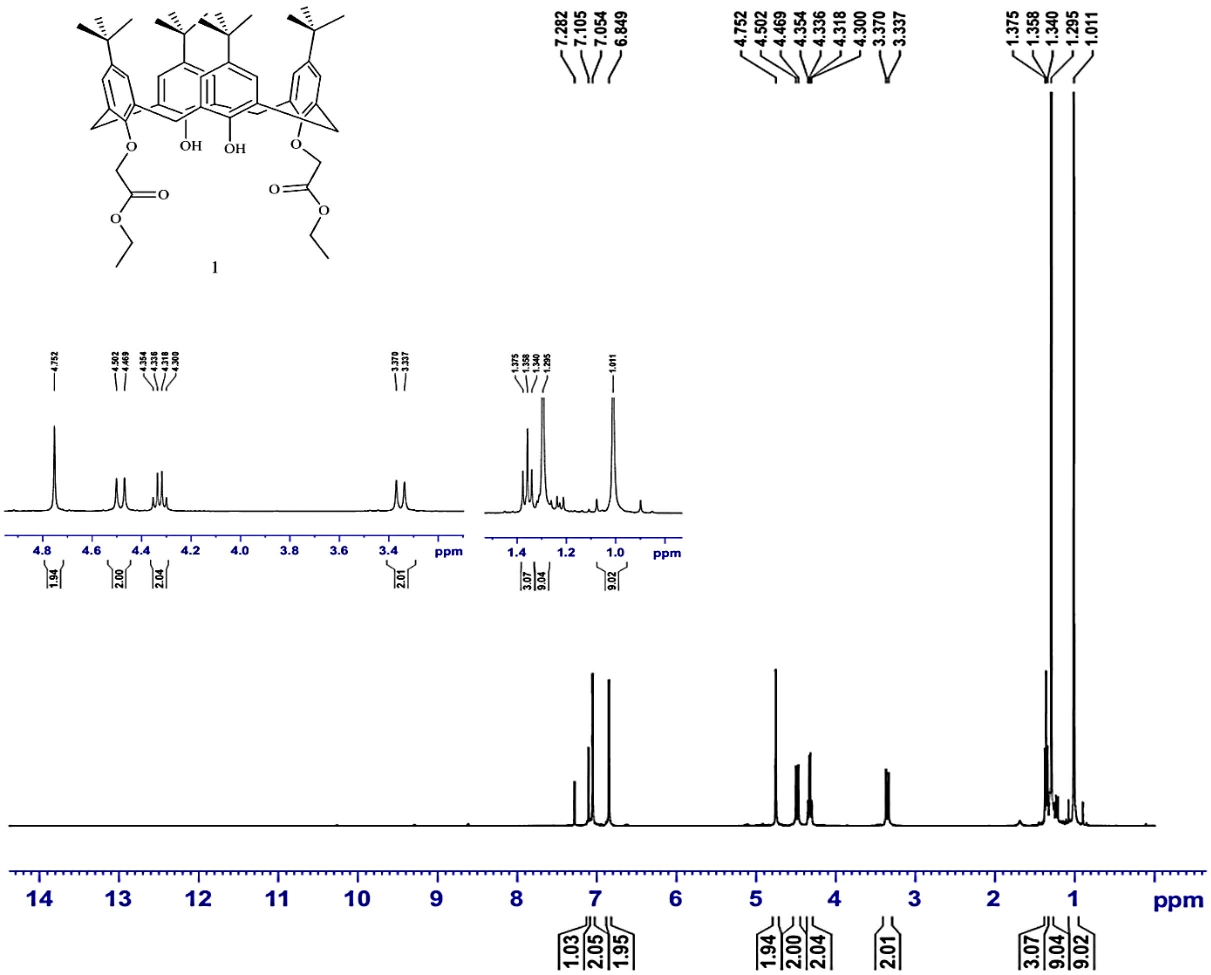

Figure S1: ^{1}HNMR (400 MHz in $CDCl_3$) of **1**.

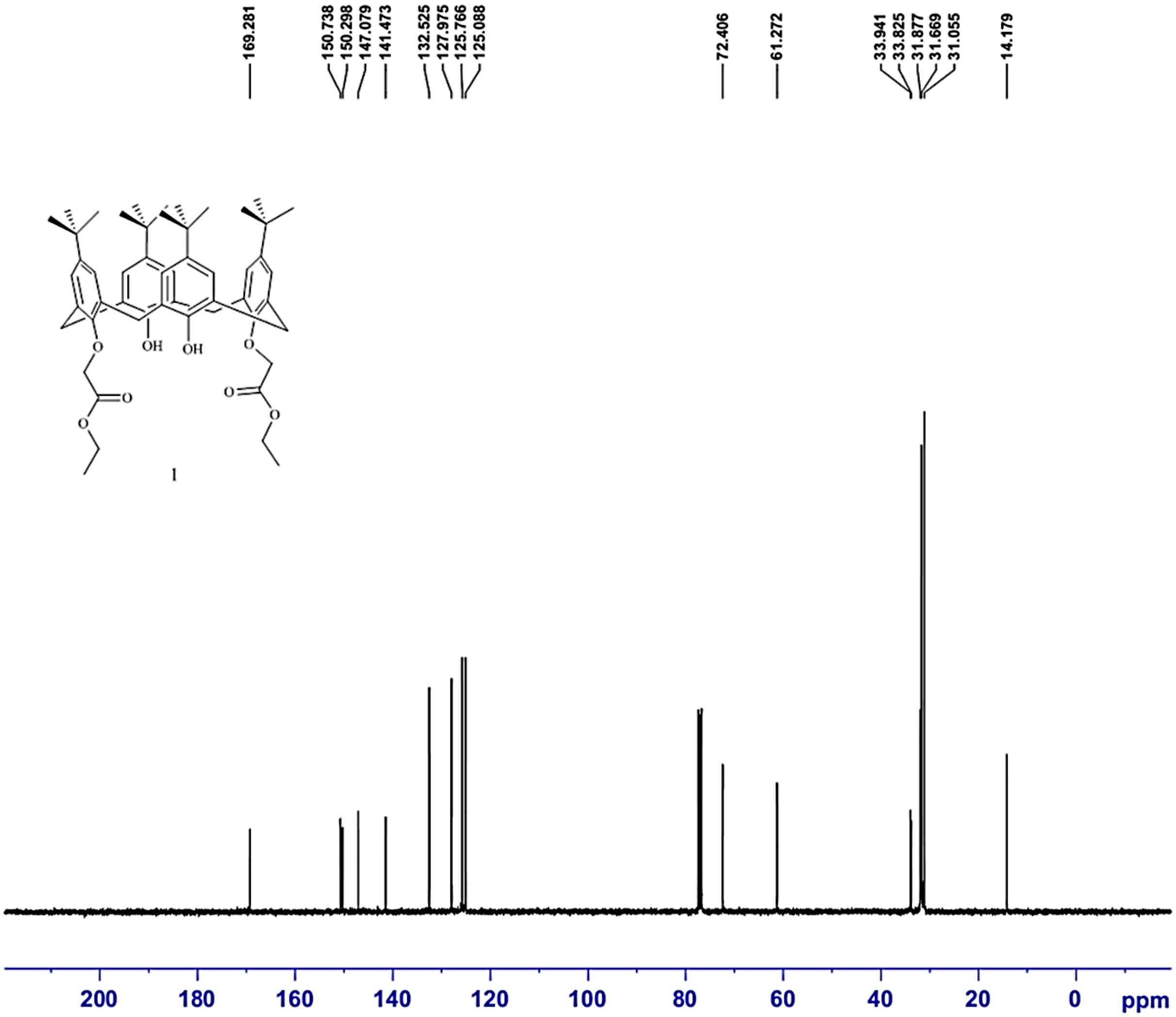

Figure S2: ^{13}CNMR (100 MHz in $CDCl_3$) of **1**.

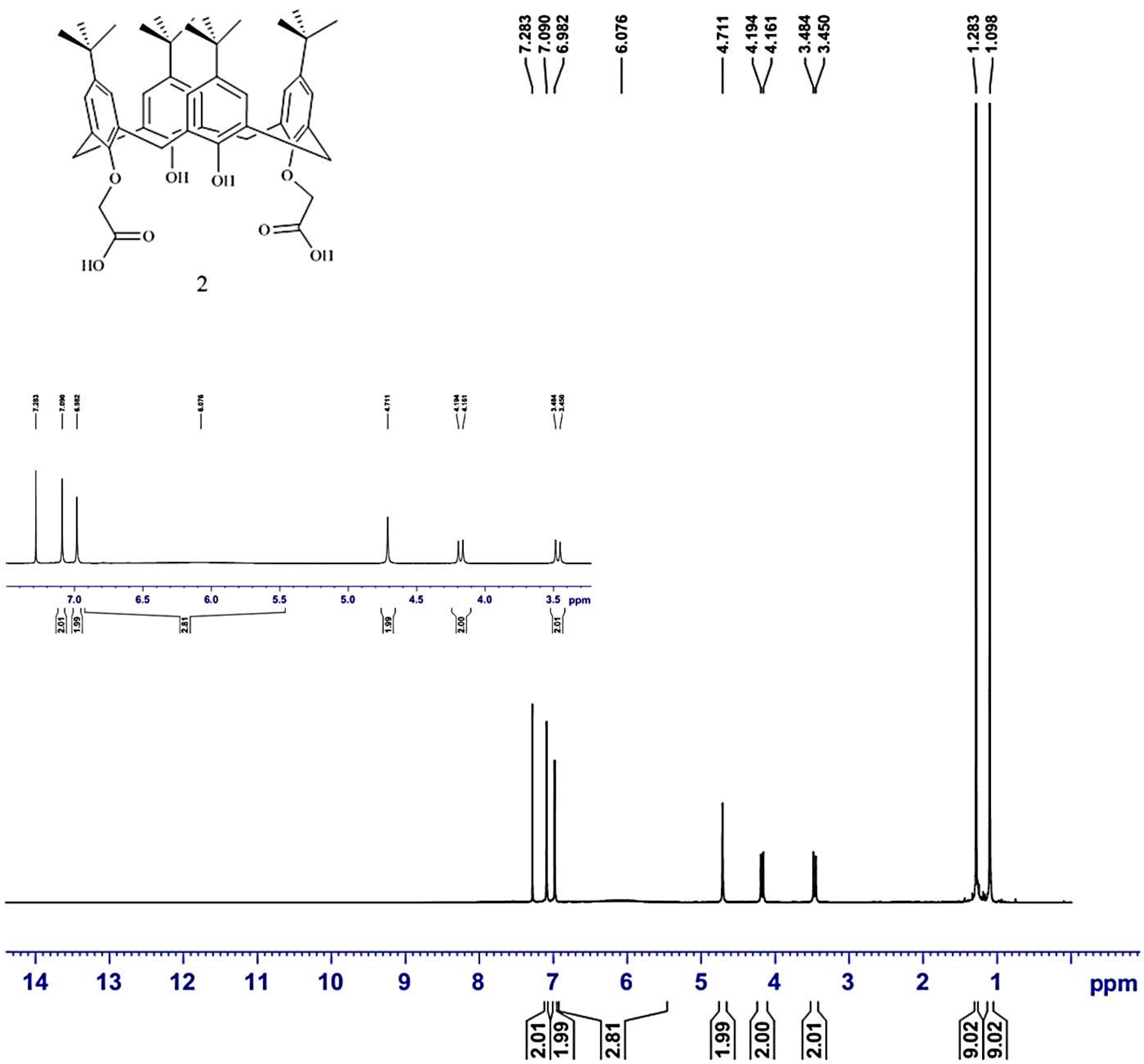

Figure S3: ^{1}HNMR (400 MHz in $CDCl_3$) of **2**.

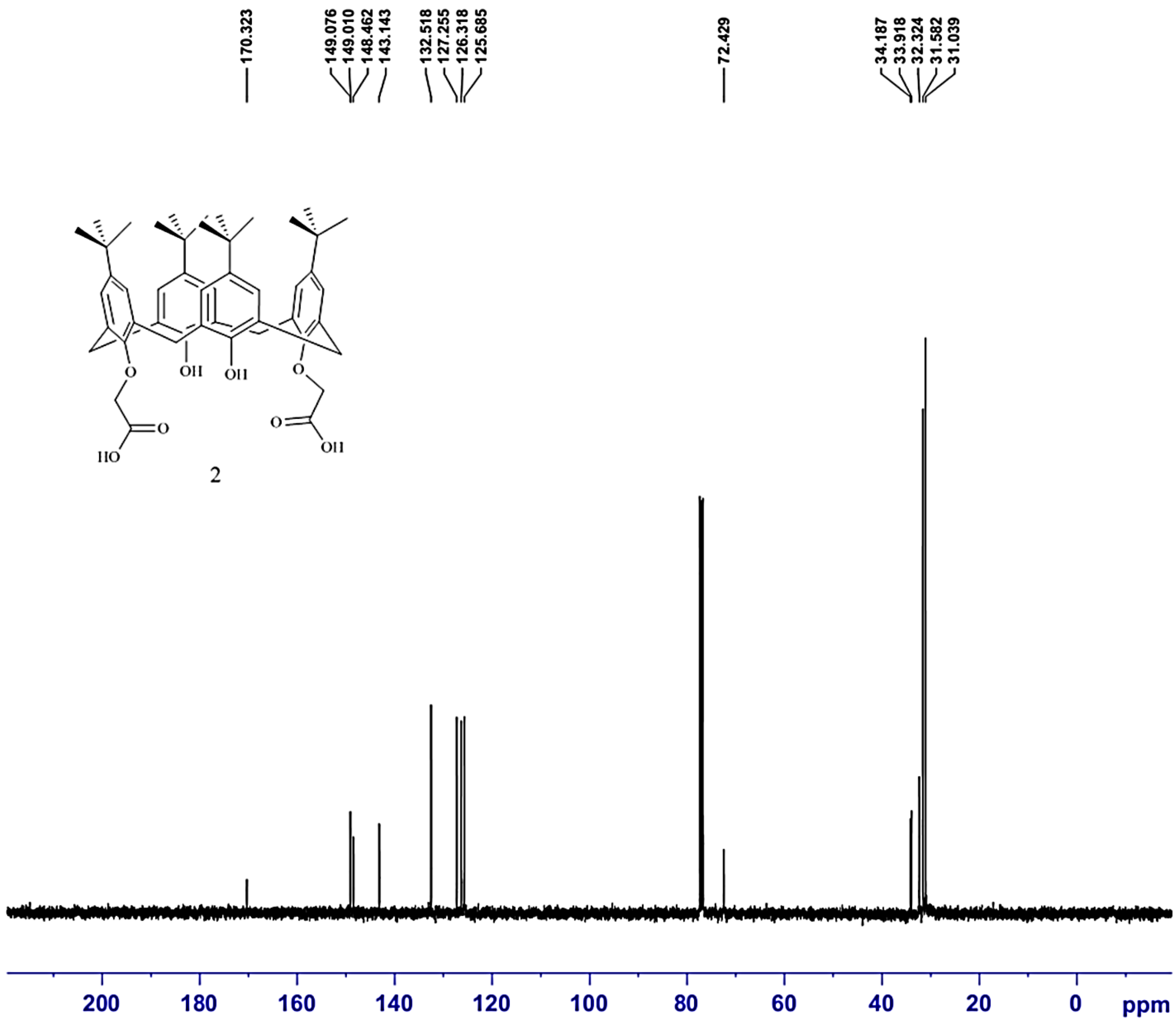

Figure S4: ^{13}CNMR (100 MHz in $CDCl_3$) of **2**.

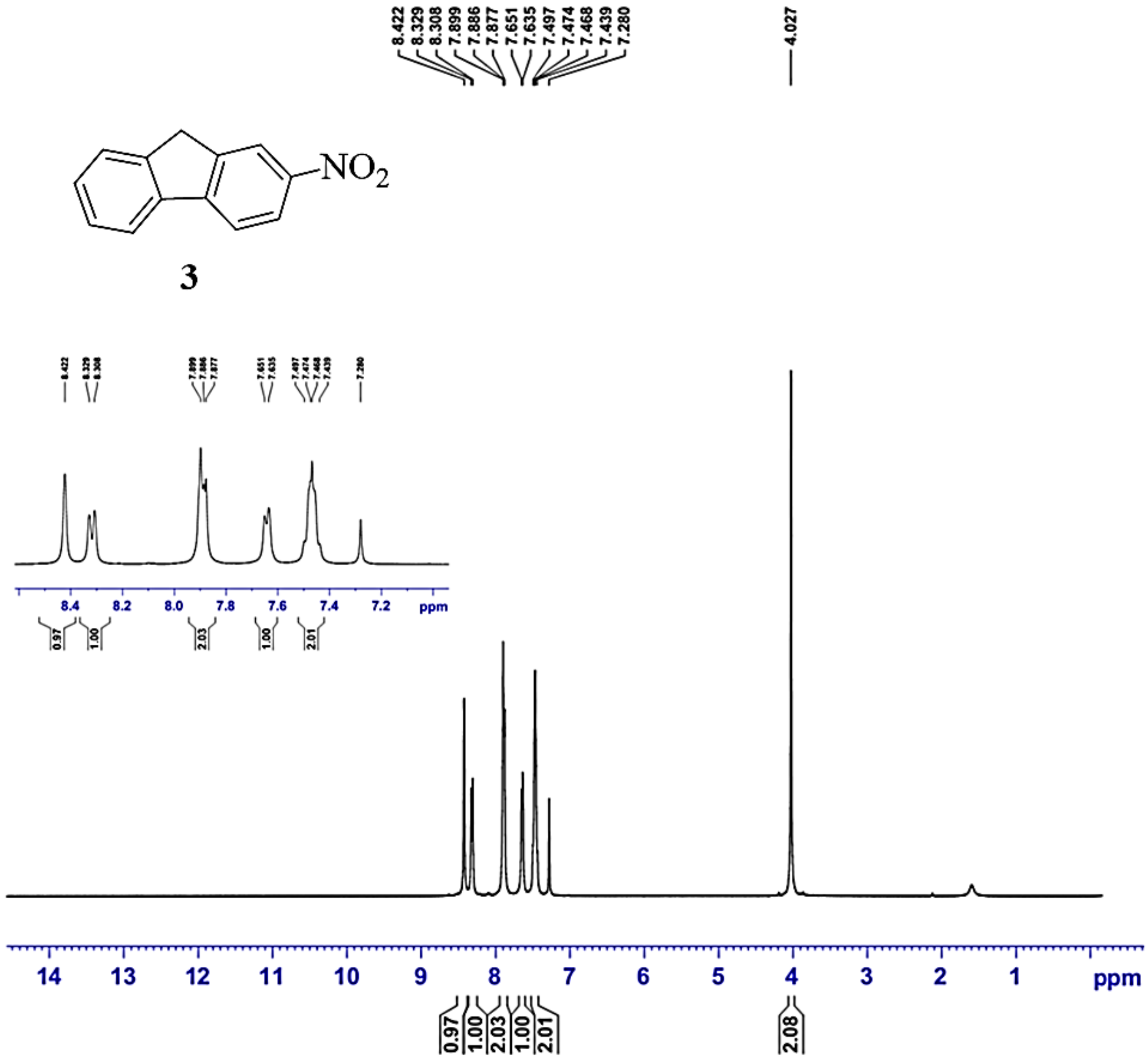

Figure S5: ^{1}HNMR (400 MHz in $CDCl_3$) of **3**.

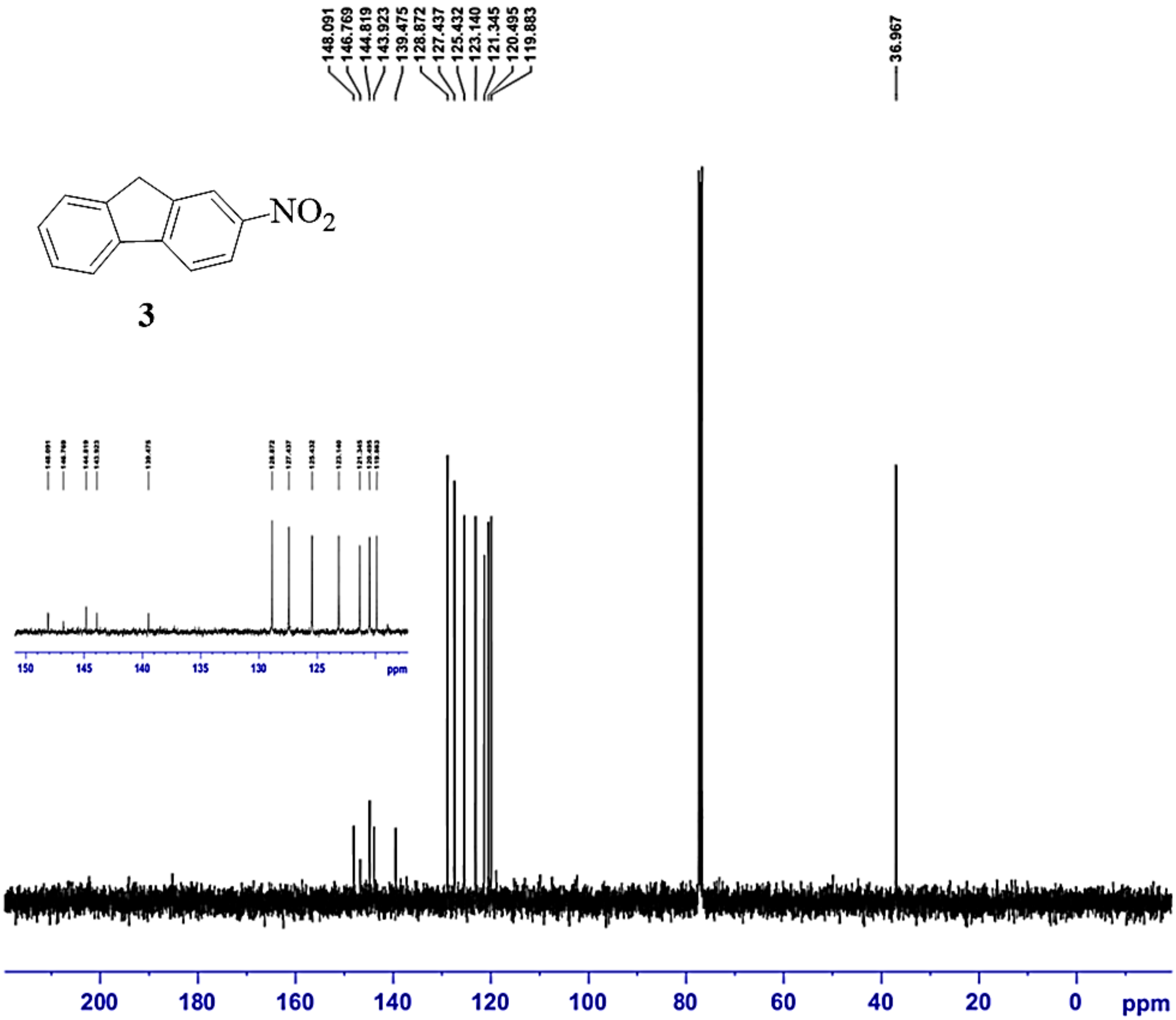

Figure S6: ^{13}CNMR (100 MHz in $CDCl_3$) of **3**.

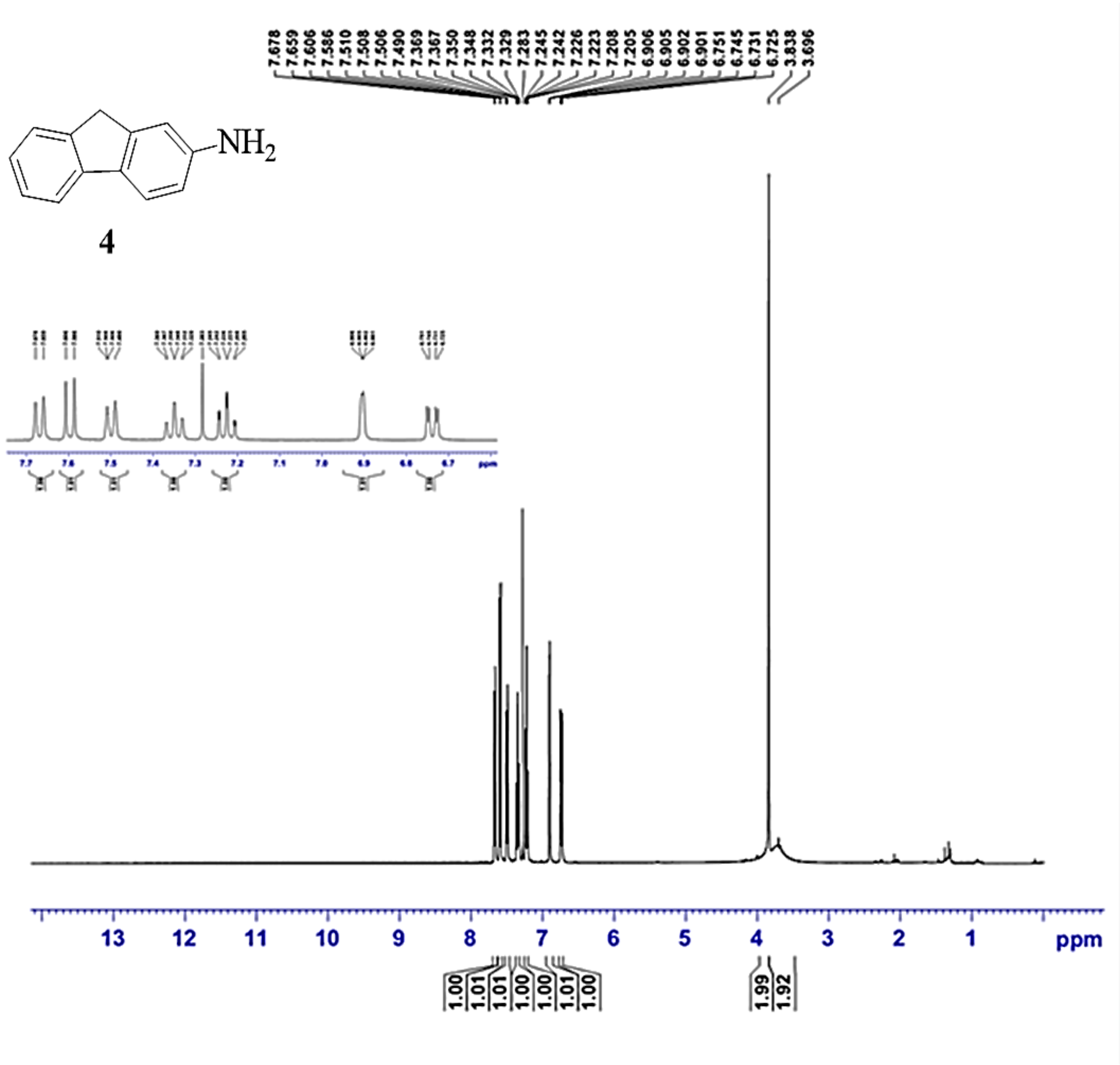

Figure S7: ^{1}HNMR (400 MHz in $CDCl_3$) of **4**.

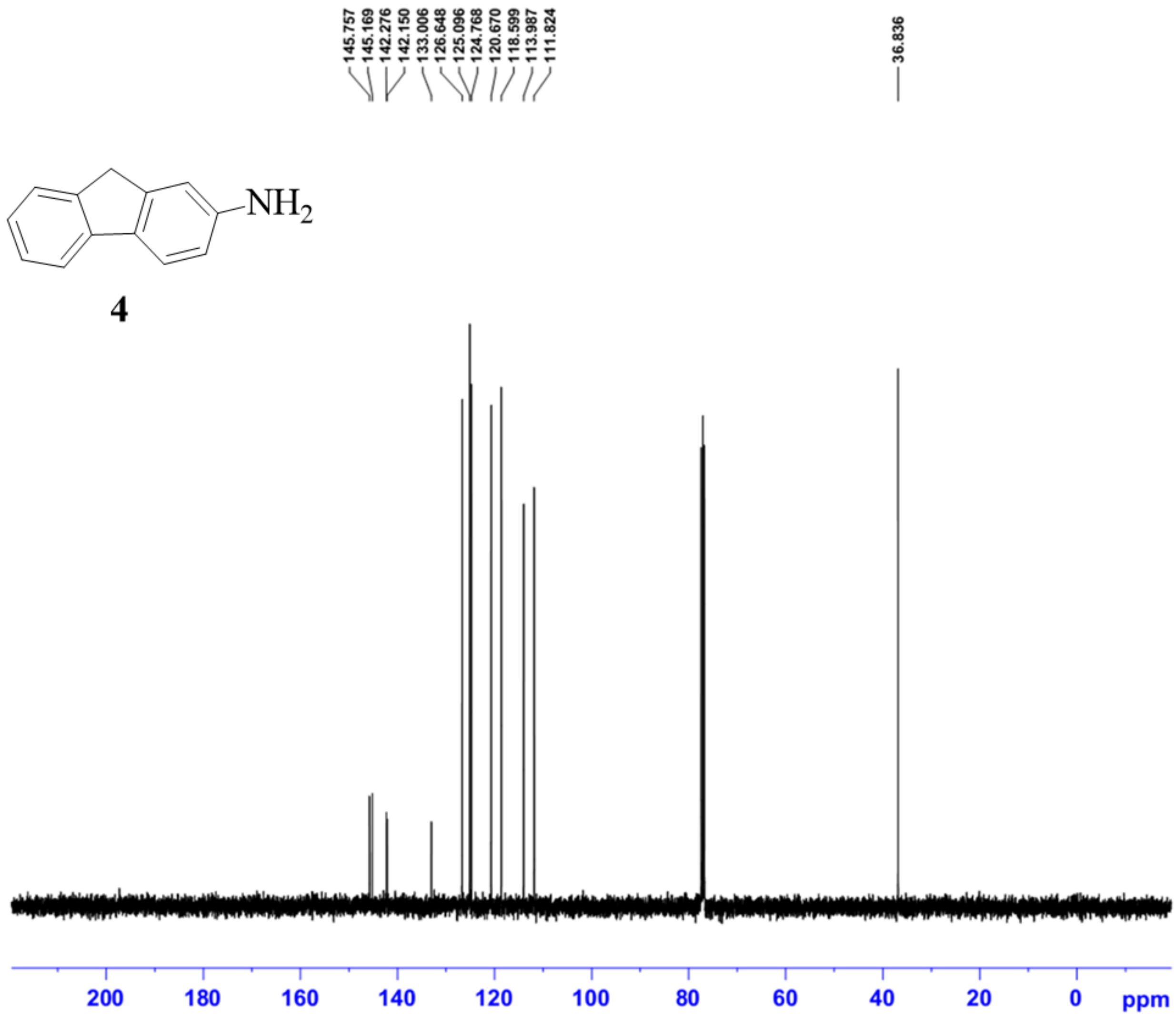

Figure S8: ^{13}CNMR (100 MHz in $CDCl_3$) of **4**.

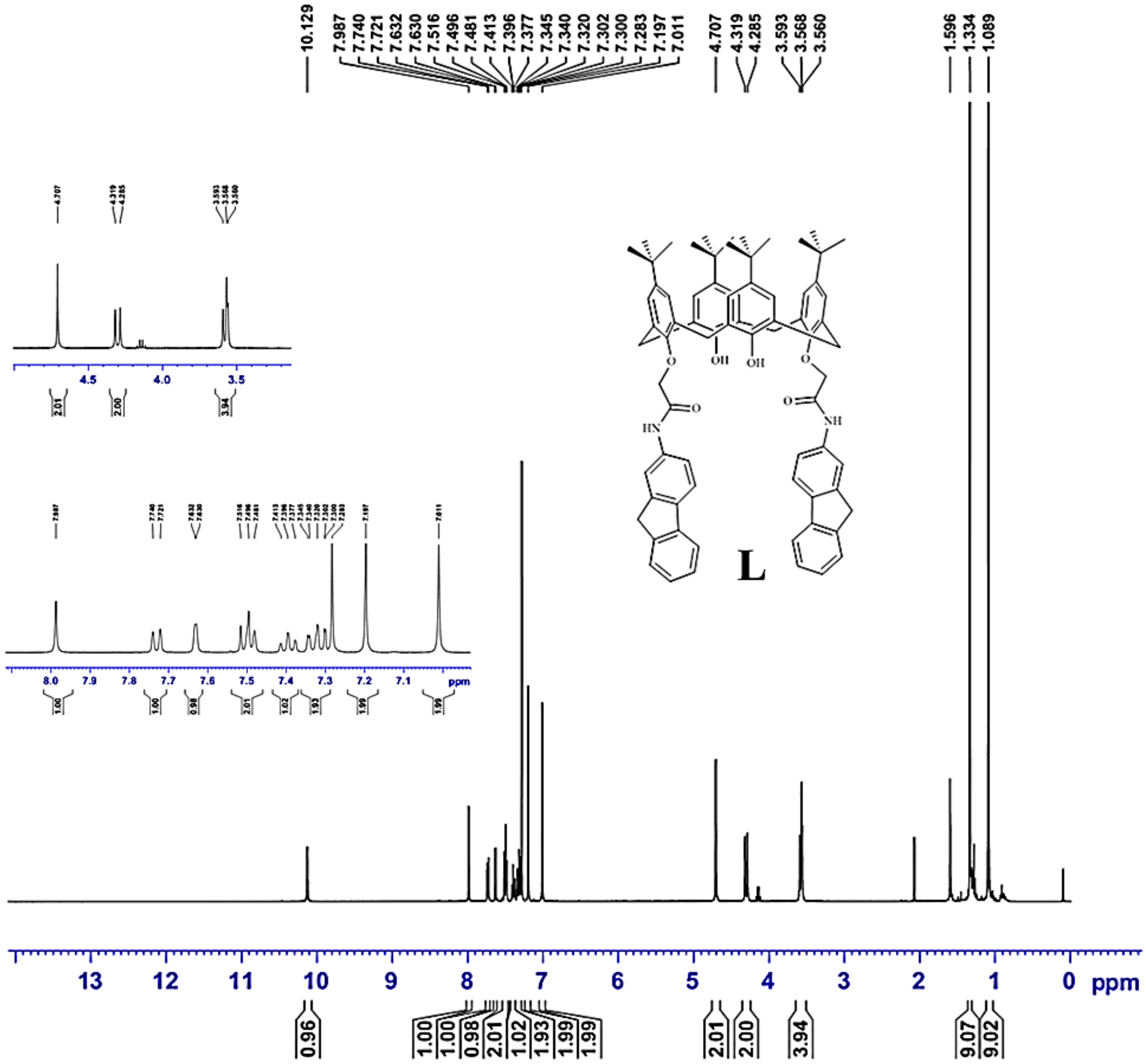

Figure S9: ^{1}HNMR (400 MHz in $CDCl_3$) of **L**.

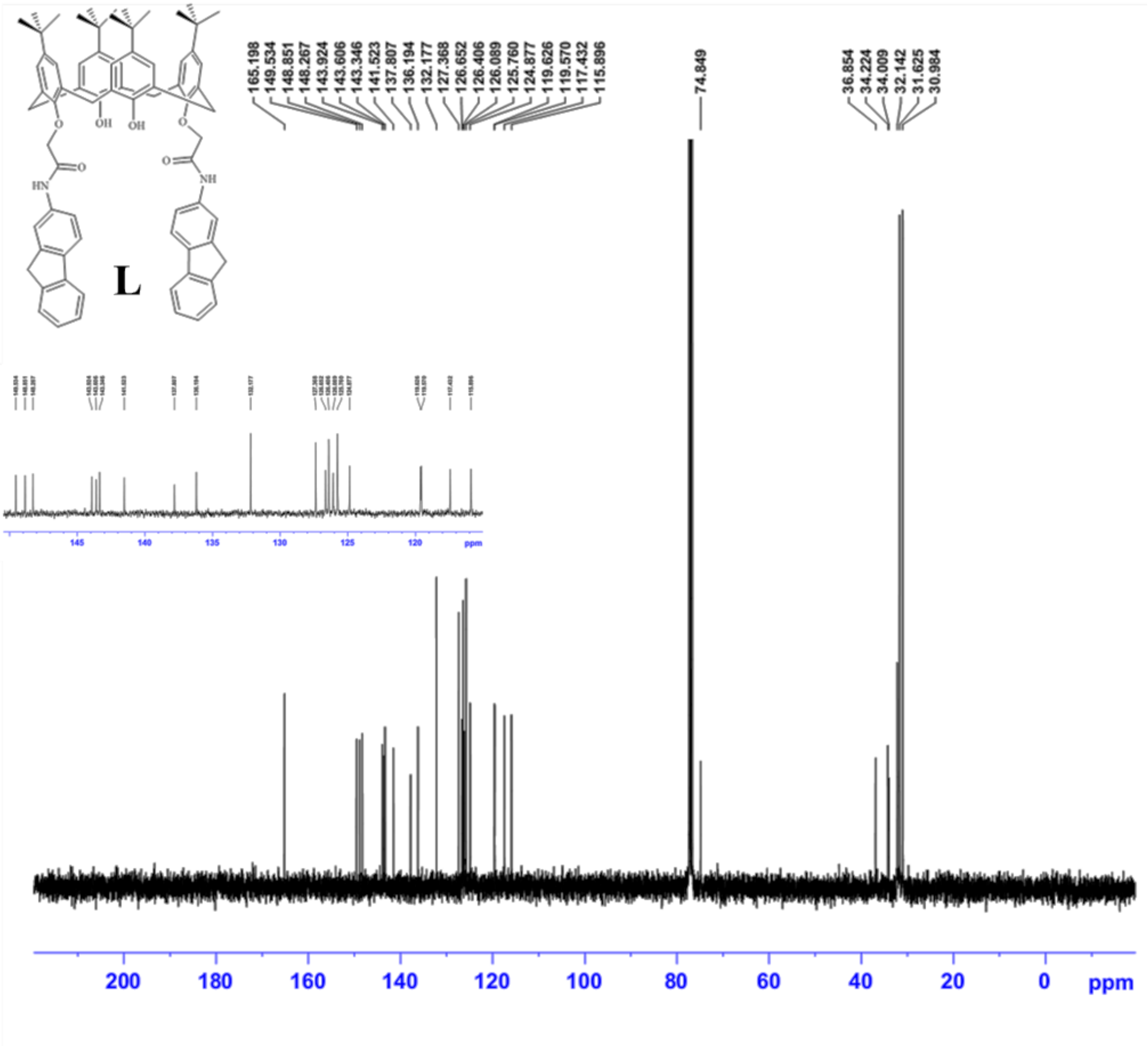

Figure S10: ^{13}CNMR (100 MHz in $CDCl_3$) of **L**.

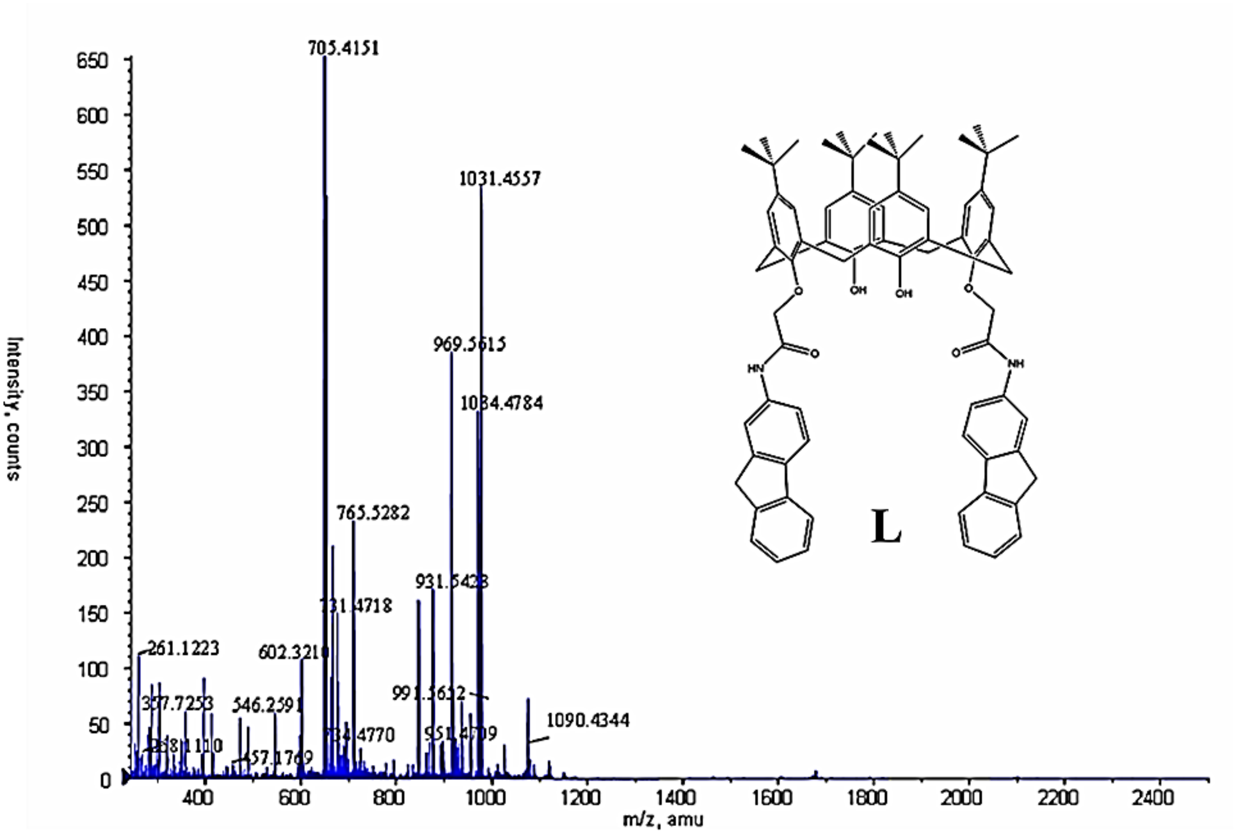

Figure S11: HRMS (m/z) of **L**.

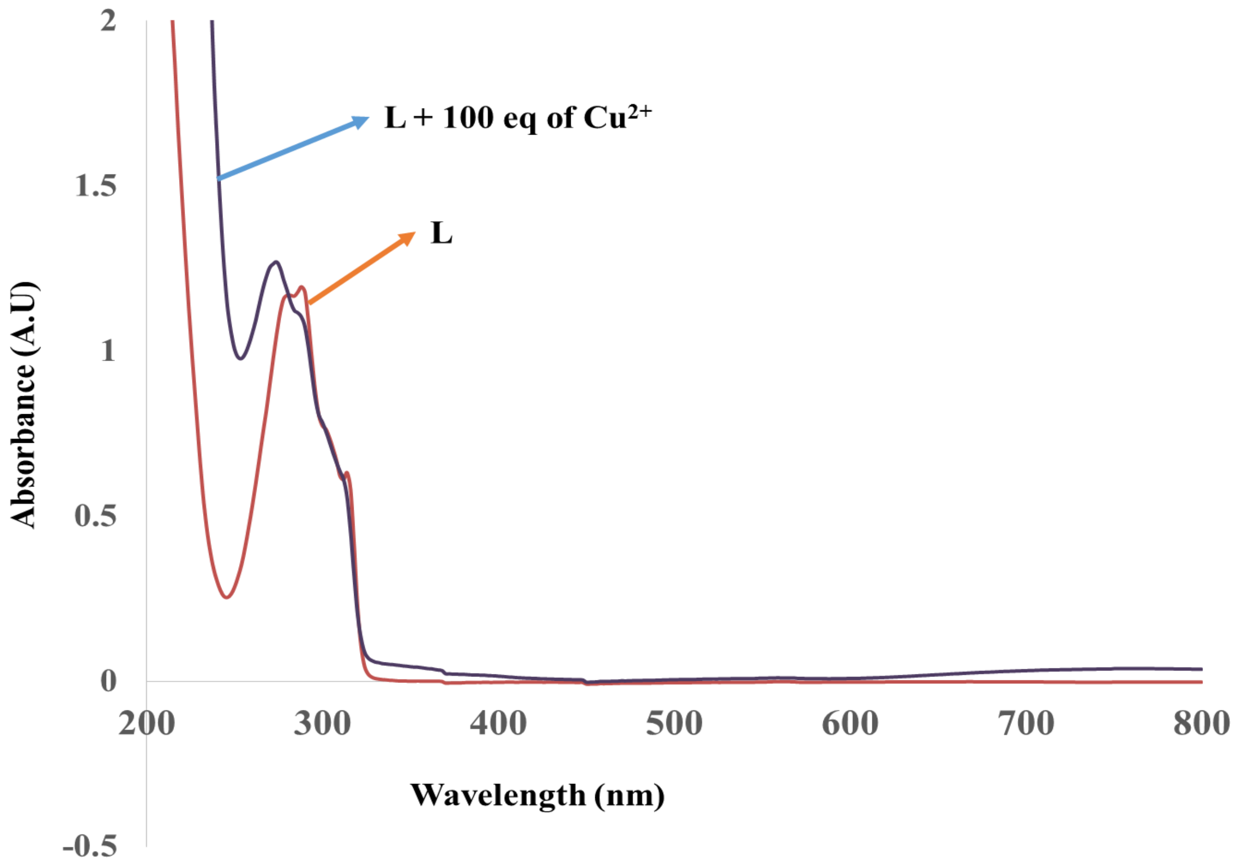

Figure S12: Absorption spectra of (**L**) (1.0 × 10^{-5}M) and Absorption spectra of **L** (1.0 × 10^{-5}M) with Cu^{2+} (100 equiv) in MeCN.

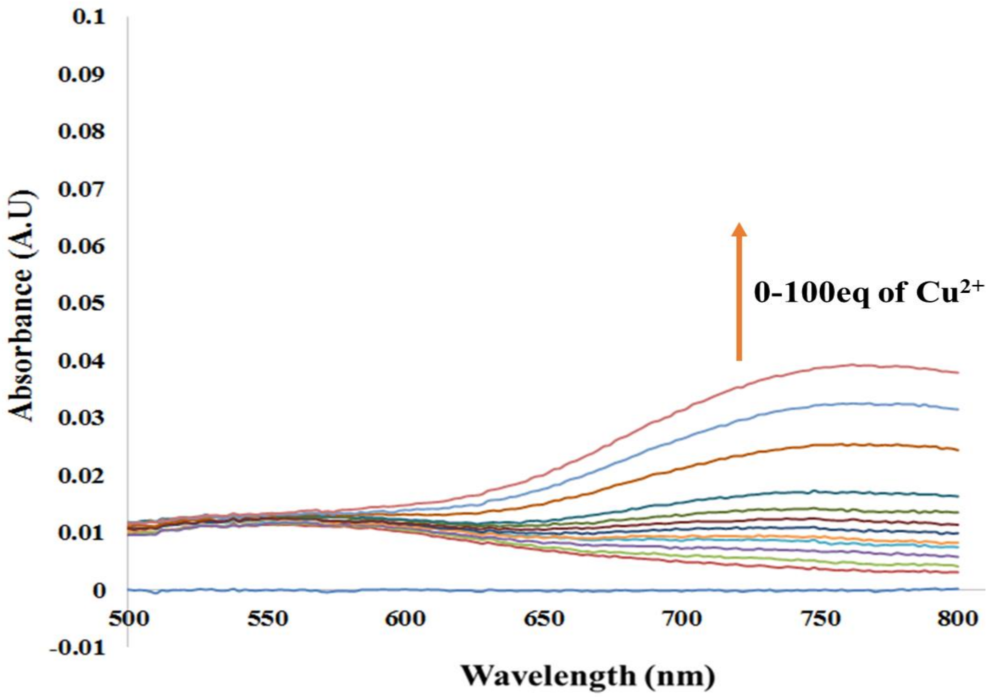

Figure S13: Absorption spectral in region 500–800 nm when **L** was titrated with varying amounts of Cu^{2+} concentration.

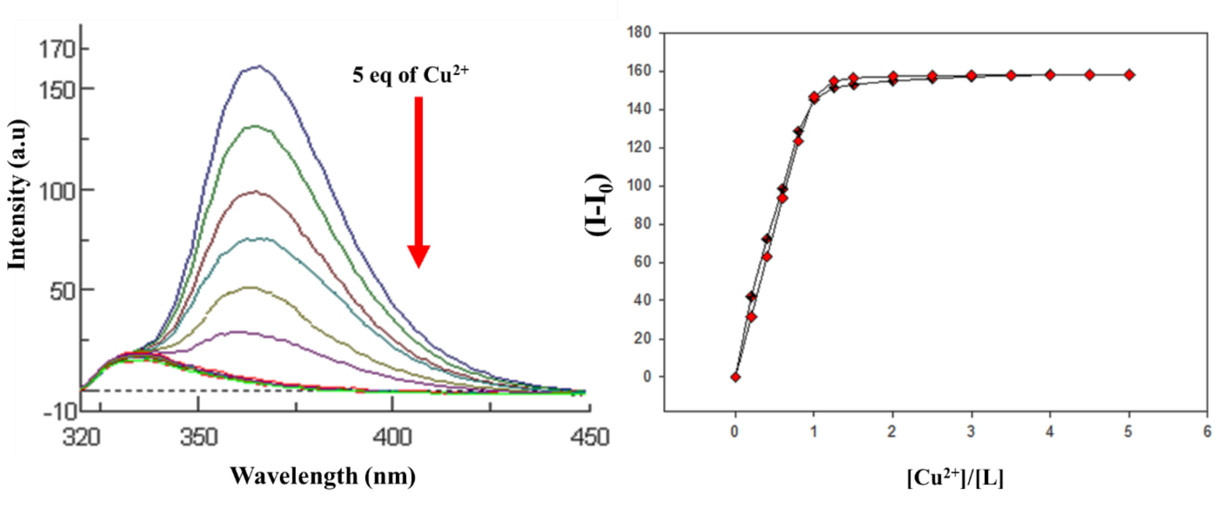

Figure S14: (a) Fluorescence spectra obtained during the titration of **L** with Cu^{2+} ion in MeCN. (b) Relative fluorescence intensity (I–I_0) as a function of [Cu^{2+}]/[**L**] mole ratio. Fluorescence intensity data for the complexes were plotted according to the Benesi-Hildebrand equation [1]: $1/(F - F_0) = 1/\{K_a \times (F_{max} - F_0) \times [M^{n+}]\} + 1/(F_{max} - F_0)$.

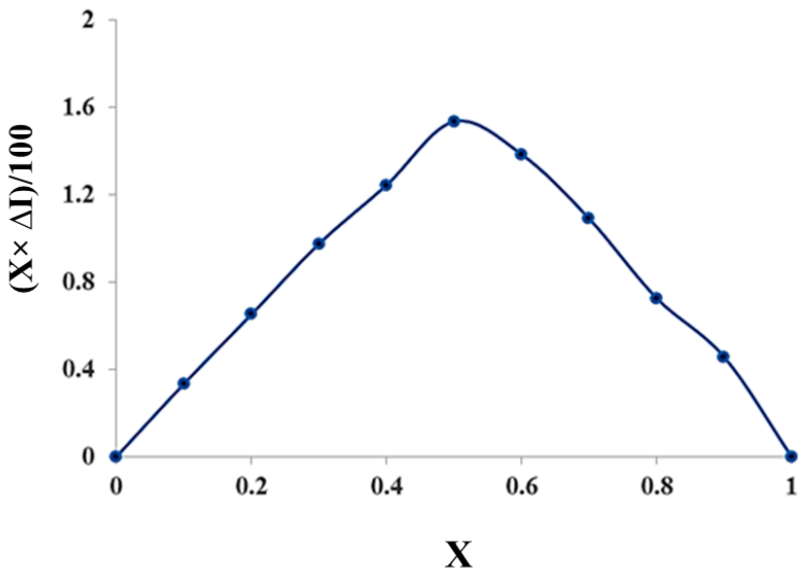

Figure S15: Job plot for determining the stoichiometry of **L** and Cu^{2+} (1.0 × 10^{-4}M) in MeCN. (X = Mole fractions, I = intensity of fluorescence).

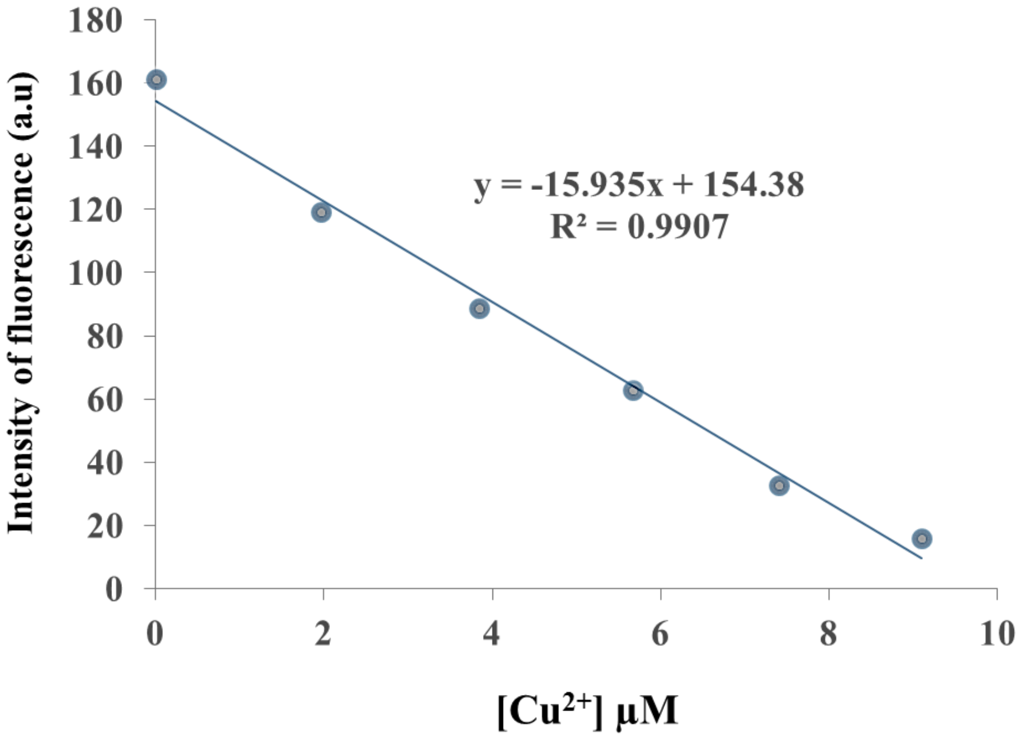

Figure S16: Calibration curve (Hill Plot) of fluorescence intensity of **L** with Cu^{2+} ion concentrations. The detection limit was determined from the fluorescence titration data based on a reported method [2,3]. The fluorescence spectrum of probe **L** was measured by 5 times repeat and the standard deviation of blank measurement was obtained. To gain the slope, the fluorescent intensity data at 365 nm was plotted as a function of the concentration of Cu^{2+}. The detection limit was calculated with the following equation:

$$\text{Detection limit} = 3\sigma/K$$

Where σ is the standard deviation of blank measurement, and K is the slope between the fluorescence versus Cu^{2+} concentration. The fluorescent intensity at 365 nm has a good linearity with concentrations of Cu^{2+} in the range from 1×10^{-6} M to 10×10^{-6} M. The linear equation was found to be $y = -15.935x - 154.38$ ($R = 0.9907$), where y is the fluorescent intensity at 365 nm and x represents the concentration of Cu^{2+} added. So the detection limit for Cu^{2+} was calculated to be 9.6×10^{-8} M (detection limit = $3\sigma/K = (3 \times 0.513)/15.935 \times 10^{-6}$).

Reference:

1. Benesi, H.A.; Hildebrand, J.H. *J. Am. Chem. Soc*. **1949**, 71, 2703.

2. Yamin Li, Y.M.; Zhang, X.L.; Zhu, B.C.; Yan, J.L.; Xu, W.P. *Analyt. Sci*. **2010**, 26, 1077.

3. Ma, Q.J.; Zhang, X.B.; Zhao, X.H.; Jin, Z.; Mao, G.J.; Shen, G.L.; Yu, R.Q. *Analyt. Chim. Acta*. **2010**, 663, 85.
